# The Impact of Technology on People with Autism Spectrum Disorder: A Systematic Literature Review

**DOI:** 10.3390/s19204485

**Published:** 2019-10-16

**Authors:** Katherine Valencia, Cristian Rusu, Daniela Quiñones, Erick Jamet

**Affiliations:** School of computer Engineering, Pontificia Universidad Católica de Valparaíso, Valparaíso 2340000, Chile; daniela.quinones@pucv.cl (D.Q.); erick.jamet@gmail.com (E.J.)

**Keywords:** user experience, accessibility, autism spectrum disorder, game-based learning, systematic literature review

## Abstract

People with autism spectrum disorder (ASD) tend to enjoy themselves and be engaged when interacting with computers, as these interactions occur in a safe and trustworthy environment. In this paper, we present a systematic literature review on the state of the research on the use of technology to teach people with ASD. We reviewed 94 studies that show how the use of technology in educational contexts helps people with ASD develop several skills, how these approaches consider aspects of user experience, usability and accessibility, and how game elements are used to enrich learning environments. This systematic literature review shows that the development and evaluation of systems and applications for users with ASD is very promising. The use of technological advancements such as virtual agents, artificial intelligence, virtual reality, and augmented reality undoubtedly provides a comfortable environment that promotes constant learning for people with ASD.

## 1. Introduction

Currently, autism spectrum disorder (ASD) affects a significant number of people who have difficulties with communication and socialization, which results in complexities for their learning. Studies have examined the use of technology and computer-based interventions to teach people with ASD language and social skills [1]. Specifically, students on the autism spectrum enjoy playing games, which provides a safe environment [2]. Thus, we reviewed the existing literature about the relationship between technology, games, user experience, accessibility, and the education and skill development of people with ASD. This article is organized as follows: Section 2 presents the theoretical background, Section 3 describes the research methodology, Section 4 analyzes the results obtained, and finally, Section 5 highlights the conclusions and recommendations for future work.

## 2. Theoretical Background

### 2.1. Autism Spectrum Disorder

Asperger’s syndrome was defined in 1944 by Hans Asperger [3]. The fifth edition of the Diagnostic and Statistical Manual of Mental Disorders (DSM-5) [4] defines autism spectrum disorder (ASD) as a condition characterized by deficits in two core domains: (1) social communication and social interaction and (2) restricted repetitive patterns of behavior, interests, and activities. Since 2013, the DSM-5 has recognized Asperger’s disorder, childhood disintegrative disorder, Rett’s disorder, and several other related disorders, as part of ASD. However, many studies still use the Asperger’s syndrome and ASD almost interchangeably.

In a study carried out by the National Institute of Health (NIH) of the USA [5] published in June 2018, it was estimated that 2.41% of children in the United States of America have an autism spectrum disorder. This shows an increase of 0.94% compared to 2010.

### 2.2. User Experience

The international standard on ergonomics of human system interaction, ISO 9241-210 [6], defines user experience as "user’s perceptions and responses that result from the use and/or anticipated use of a system, product or service". In other words, the user experience is the degree of "satisfaction" that the end user has with the system or service after using it, that is based on each of the interactions that he or she has.

According to Peter Morville [7], user experience is meaningful and valuable when a product, service or system is useful (that is, its content is original and satisfies a need), usable (the product is easy to use), desirable (the image, identity, brand, and other design elements produce positive emotions towards the product), locatable (the content is accessible to people with disabilities), credible (users have confidence in the product), and valuable (an added value is generated from the product).

### 2.3. Accessibility

The international standard on ergonomics of human system interaction, ISO 9241-171 [8] defines accessibility as the “extent to which products, systems, services, environments, and facilities can be used by people from a population with the widest range of user needs, characteristics and capabilities to achieve identified goals in identified contexts of use”. In other words, accessibility is the condition that environments, services, processes, and objects (everything that involves an interaction) must meet, which must be understandable and usable by the broadest range of people, regardless of their capabilities.

### 2.4. Game-Based Learning

Games that use technology are widely used to teach people conceptual knowledge and skills. There are different implementations of such games, such as serious games, gamification, and e-learning.

#### 2.4.1. Serious Games

Serious games are games whose main objective is not fun or entertainment but the learning or practice of skills. In 1970, Clark Abt [9] defined this concept as follows in his book called “Serious Games”—“games that have an explicit and carefully thought-out educational purpose and are not intended to be played primarily for amusement. This does not mean that serious games are not, or should not be, entertaining”.

#### 2.4.2. Gamification

The concept of gamification was developed in 2003, and its use became widespread in 2010 through the work of multiple professionals. Gamification is formally defined as "the use of game elements and game design techniques in nongame contexts" [10]. When we talk about gamification, we tend to interpret it as a methodology where the purpose is to provide rewards to users to inspire personal and collective commitment, but this interpretation is very far from reality. Many authors maintain that the success of a gamified system or process lies in good design and adequate feedback, among many other factors. Other authors have supported this argument: for example, Kapp [2] stated, "Do not think of gamification as only the use of badges, rewards, and points. Instead, think of the engaging elements of why people play games—it is not just for the points—its [sic] for the sense of engagement, immediate feedback, and the success of striving against a challenge and overcoming it".

#### 2.4.3. E-Learning

The term “e-Learning” comes from the abbreviation of “electronic learning”. Khan [11] defined e-Learning as "a hypermedia instructional program that uses the attributes and resources of the Internet to create meaningful learning environments." That is, e-Learning refers to online teaching and learning through the Internet and technology.

### 2.5. Game Elements 

Game elements are the components that make up a game to create an attractive experience for players. Werbach [10] described 25 such game elements. For the purpose of our study, we identify the relevant game elements are as follows:

- Narrative: Telling of a coherent story.
- Progression: Player growth and development.
- Challenges: Tasks that require an effort to perform.
- Competition: Players or groups that win or lose.
- Rewards: Benefits granted after a certain action.
- Feedback: Information about how the player is performing.
- Avatars: Visual representation of a player character.
- Collections: Set of items that can be accumulated.
- Levels: Steps defined in the progression of a player.
- Leaderboard: Visual representation of the player’s progression with respect to others.
- Points: Numerical representation of the player’s progression.
- Achievements: Accomplishment of defined objectives.
- Teams: Group of players who work together to achieve a common goal.

## 3. Research Methodology 

This systematic literature review was carried out following the process proposed by Kitchenham [12]. Kitchenham outlined three fundamental phases for conducting a review of the literature: (1) planning the review, which includes creating the research questions and reviewing the protocol; (2) conducting the review, which includes the review, the selection and quality of studies, data extraction and data synthesis; and (3) publicizing the results after the review. Next, we detail the process followed for this document.

### 3.1. Research Questions

To cover every topic of interest in this systematic literature review, we formulated three research questions. These questions consider relevant and general aspects important for comprehending the concepts that we think are important for this study. These questions can be seen in Table 1.

### 3.2. Data Sources and Search Strategies

To conduct this systematic literature review, we searched for scientific papers on five databases: IEEE Xplore Digital Library, ACM Digital Library, Science Direct, Scopus, and Web of Science. For these sources, we considered only documents that were relevant in computer-related categories, such as technology, engineering and computer science, excluding categories related to medicine or chemistry. Additionally, we selected articles published during the last 10 years, between January 2009 and June 2019.

### 3.3. Article Selection

Once we chose the databases to search, we determined the specific search strings to find articles to answer the research questions and defined the exclusion and inclusion criteria to refine and filter the articles found.

#### 3.3.1. Search Strings

We formulated the search strings based on the relevant topics to our systematic literature review. We determined a set of specific keywords to use in our queries, i.e., “Autism Spectrum Disorder”, “Accessibility”, “User Experience”, “Gamification”, “Serious Games”, and “Game Elements” that would be useful to answer our research questions.

These strings were focused on finding studies that analyzed or experimented with the use of games with people with ASD, considering aspects such as the user experience, accessibility, and game elements. In Table 2, we present the specific search strings that were used in the selected databases.

#### 3.3.2. Study Selection Criteria

To answer the research questions based on the selected articles and develop a general knowledge of the concepts that we were working with, we included the conditions listed in Table 3.

The types of papers presented in Table 4 were excluded.

### 3.4. Document Selection

**Figure 1:**
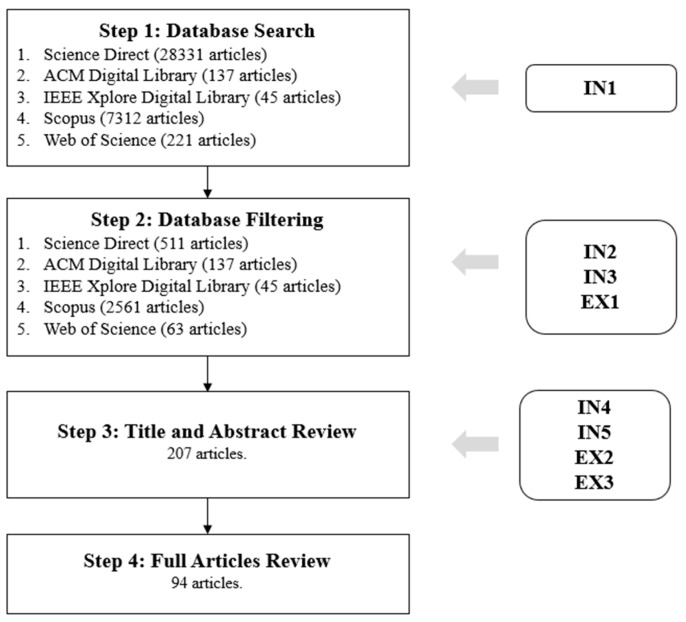
Flow chart with the results of the article selection process.

Applying the selection criteria, we gathered a total of 94 articles. Figure 1 shows the general process flow of the search and study selection for this review, detailing the inclusion and exclusion criteria applied in each step.

### 3.5. Data Synthesis

After the search, we extracted the information from each of the 94 studies, summarizing and tabulating the information based on different metrics, such as the year published, document type and paper category. In the following steps, we detail each of the metrics.

#### 3.5.1. Year of Publication

**Figure 2:**
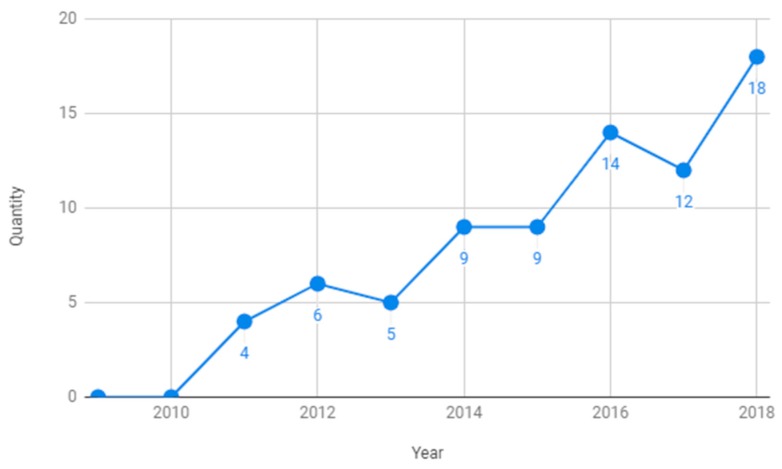
Year of publication.

As detailed above in the inclusion criteria section, we considered studies published during the last 10 years, between 2009 and 2019. As shown in Figure 2, we plotted the number of studies that were found that were published between 2009 and 2018, and we observed an increase in publication on this topic over this period. The studies found in 2019 are not presented in this plot because it would have been misleading to show incomplete data, as this review was finished in June 2019. Seventeen studies published in 2019 were found (almost equal to the number of publications in 2018), which led us to believe that this number will undoubtedly increase significantly during the remaining months of 2019.

#### 3.5.2. Document Type

**Figure 3:**
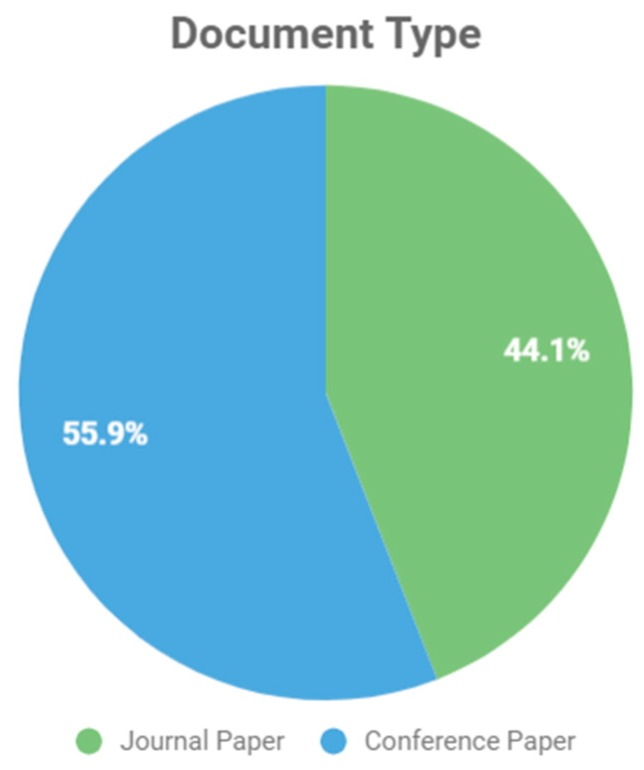
Document Type.

We analyzed the origin of the studies reviewed and determined whether they were conference proceedings or had been submitted to a scientific journal. Figure 3 shows a relative balance between the number of papers that were published as conference proceedings and in journals.

#### 3.5.3. Document Categories

The studies were categorized as follows:

- Review: An updated summary of a particular topic is provided.
- Case Study: A solution is given to a presented problem based on a tool, methodology, etc.
- Empirical Data: A context or situation is analyzed based on historical data.

**Figure 4:**
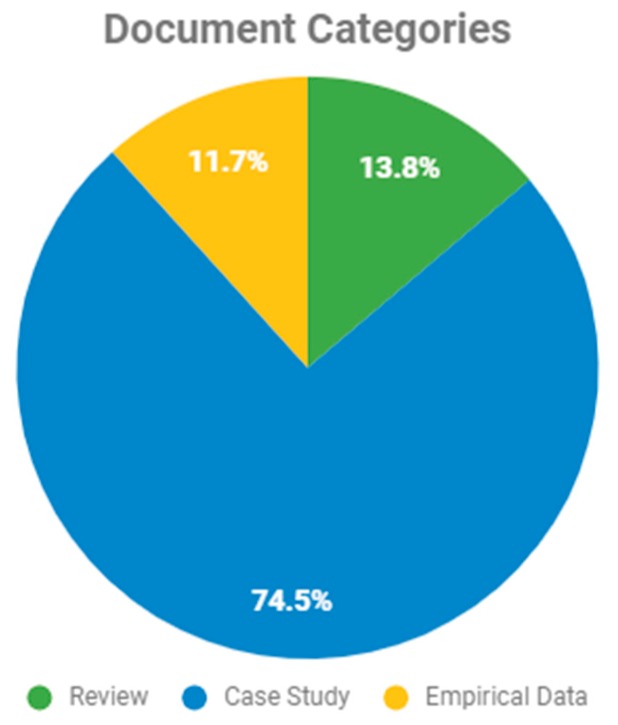
Document Categories.

Figure 4 shows that 74.5% of the studies analyzed were case studies. It is believed that this is because the researchers were focused mainly on conducting investigations and accomplishing their study objectives, such as teaching conceptual skills.

## 4. Results and Discussion

After applying each of the filters described in the “Study Selection Criteria” section, as shown in Figure 1, a total of 94 studies were obtained. These studies were analyzed under different metrics, as seen in the “Data Synthesis” section. Based on our review of these studies, we now answer our research questions, considering those studies that are relevant to the specific context of each question.

**RQ1.**  **In what way does the use of technology contribute to the education of people with autism spectrum disorder?**

As mentioned in the previous sections, ASD is a condition that is categorized as a disability due to the cognitive disorders that people with ASD face [13]. Several studies showed that most people with autism show a natural affinity for technology and a good disposition for using technology and learning through the use of computers [14]. This is because the environment and context that these experiences provide are predictable and structured, which helps people with ASD to maintain their routines and repetitive behaviors without affecting their comfort [15].

Several studies proposed the use of modern technologies to help teach skills to people with ASD. Some interesting examples of new technological approaches are the use of sensors, virtual reality, virtual agents, augmented reality, geolocation, and Kinect, as presented in the following studies. Wojciechowski et al. [15] developed a mobile application that, in conjunction with the use of Estimote Beacon sensors to identify objects, supports children with ASD in pronouncing new words and identifying their meanings. Lorenzo et al. [16] proposed an application that uses virtual reality and robots with cameras to detect children’s emotions, adapt system interactions and thus develop social skills in students with autism spectrum disorder. Bernardini et al. [17] presented ECHOES, which is a serious game that focuses on the development of activities to promote social communication in children with ASD using an autonomous virtual agent that acts as a companion for children during their interactions with the system. Sorce et al. [18] developed an exploratory study to evaluate the effectiveness of the use of Kinect as a tool to allow people with ASD to explore works of art in a touchless virtual environment and assess whether this generates greater interest in them. Escobedo et al. [19] presented the Mobile Social Compass (MOSOCO) application, which makes use of augmented reality through a mobile device camera to include game elements in real social situations with the aim of developing social skills in children with ASD. Silva et al. [20] presented a serious game that, through geolocation, virtual reality and augmented reality, creates a virtual environment with 3D virtual monsters positioned all over the world that aim to teach children with ASD relevant educational content, such as vocabulary.

In addition to examining the studies from a technological perspective, we categorized the 94 studies based on the following learning topics with the goal of understanding the contribution of technology to education for people with ASD in terms of the specific skills that they focus on teaching: Conceptual Skills (subtopics: Language, Money, Colors, Mathematics, Programming, and Science), Practical Skills (subtopics: Health, Daily Life, and Transportation), Social Skills (subtopics: Communication, Emotions, and Interpersonal Relationships) and General Skills (subtopic: General). Table 5 shows the percentage of studies for each of the topics and subtopics, and in the same way, Table A1 (available in Appendix A) details each of the topics and subtopics according to which the articles were categorized. The results obtained after categorizing the studies are presented in the following sections.

### 4.1. Conceptual Skills

First, 25.53% of the studies focused on analyzing and fostering skills within the range of Conceptual Skills. Studies in the Language subcategory focused on promoting the learning of expressions, thoughts and feelings through words. Examples of this include studies [13,21]. Arciuli and Bailey [13] analyzed a small group of children with ASD that were literate using the ABRACADABRA application and observed significant improvements in reading accuracy in participants who interacted with the system but not in children who did not use the application. For the children who did not use the application, their lack of improvement was believed to be due to their lack of socialization aspects that children must exhibit when interacting with a teacher to develop reading ability. Khowaja et al. [21] developed a prototype of a serious game for children with ASD to learn vocabulary. The effectiveness of the game was assessed through the comparison of children’s performance at the beginning of the intervention, after the use of the prototype and 1–2 weeks after the use of the prototype, which enabled the researchers to track the improvement in the children’s vocabulary.

Another subcategory of Conceptual Skills is the Money subcategory and only one study [22] was assigned to this subcategory. Caria et al. presented the design of a game that helps people with autism spectrum disorder acquire skills to help them understand the concept of money and its applications in real life, which was tested by obtaining positive and promising results.

In addition, like the Money subcategory, the Colors subcategory also included only one study, [23]. In this study, based on cognitive theories, Tuğbagül et al. developed a computer interface for students with ASD and mild mental disability that used their preferred colors and helped them maintain their concentration.

Additionally, the studies in the Math subcategory aimed to develop skills related to numbers. Examples of studies in this subcategory are [24,25]. Tashnim et al. [24] developed the Play and Learn Number (PLaN) application, which teaches arithmetic and calculus to children who have ASD and helps children memorize and recognize numbers (in or not in sequences) through animated images. Muñoz-Soto [25] developed an application to support professionals in teaching functional mathematics and calculus to children with ASD. Through tests, it was possible to demonstrate that this application promotes the development of mathematical skills. However, it was suggested that the application should be tested by more users and in different institutions.

The Programming subcategory included studies that aimed to develop skills related to computational programming, for example, to design and order actions and commands. Only two studies were assigned to this subcategory, i.e., [26,27]. Eiselt and Carter [26] planned and conducted programming classes through Scratch for children with ASD with the aim of developing their technical and social skills. Despite their efforts, no real evidence of an increase in students’ social learning or behavior was found. However, while the students did not develop social skills as expected, the authors suggested that the students knew more about programming after the experiment since at the beginning, they did not have any notion of programming, but after the experiment, they could read and write processing programs. Schmidt and Beck [27] proposed a learning intervention based on digital games for young people with ASD to develop their social skills as they worked on teams to solve introductory computer programming problems with virtual and programmable robots. According to the authors, this intervention has the potential to help participants develop social skills, however, because this study was only concerned with the initial stages of development, there was no analysis of the data, so conclusions regarding cognitive skills could not be made with certainty.

Finally, the studies in the Science subcategory investigated and interpreted natural, social, and artificial phenomena. For this subcategory, we found only one study [28], in which Eder et al. developed a mobile game application as a complementary learning material to teach children with ASD parts of the human body. After the intervention, it was observed that the application was very useful for teaching and that the motivation levels of the participants increased significantly.

### 4.2. Practical Skills

Second, the Practical Skills category included only 8.51% of the identified studies and was subdivided into several subcategories. First, the Healthcare subcategory concerned teaching about the health care that people should have. An example of a study in this subcategory is [29]. De Urturi et al. [29] developed a system consisting of a set of serious games aimed at teaching first aid (such as what to do in certain situations and basic knowledge about medical care and medical specialties) to people with ASD. Because the application was still in development, only partial results were available, so to determine if these results were promising, the authors administered a simple questionnaire to the participants, as they obtained positive results, they decided to continue developing the project.

Another subcategory of Practical Skills is the Daily Living subcategory. The studies in this subcategory focused on building knowledge about the development of daily recurring activities, and examples are [30,31]. Pérez-Fuster et al. [30] analyzed the impact of an intervention with digital technology (DT) compared to that of a treatment-as-usual (TAU) intervention on adults with ASD. The DT intervention sought to improve daily life skills, such as washing dishes and washing clothes. The results showed that the DT intervention significantly improved the daily life skills of the participants and was more effective than the TAU intervention. Santarosa and Conforto [31] presented a tablet application for children with ASD and children with intellectual disability (ID) that seeks to teach and develop routines in the classroom and verbal communication by directly involving teachers and assistants in schools. Children with ASD successfully adapted to the application, and their socioadaptive behaviors both in the classroom and related to verbal communication improved greatly. On the other hand, children with ID did not achieve autonomous use of the application, and they only had improvements in nonverbal classroom routines.

The final subcategory within the Practical Skills category is the Transportation subcategory. The studies in this category were concerned with teaching the necessary knowledge that individuals need to be able to transport themselves effectively. Some examples of this are found in [32,33]. McKissick et al. [32] investigated the impact of a computer instruction package to teach map-reading skills to three elementary students with ASD. Very promising results were obtained for interventions that used technology with children with ASD, such as increased levels of learning and improved learning habits among students. De Los Rios [33] proposed a draft of a study to evaluate platforms and interfaces that help users transport themselves, such as Google Maps or Apple Maps with eye tracking. They compared these platforms and interfaces with a proposed system that would provide a more personalized environment that is adapted and accessible to the needs of people with ASD.

### 4.3. Social Skills

Third, the Social Skills category included 36.17% of the total resulting studies and was subdivided into three subcategories. The studies in the first subcategory, Communication, focused on the development of skills such as exchanging information between two or more individuals and examples from this subcategory are found in [34,35]. Milne et al. [34] investigated the use of autonomous virtual humans (self-directed) to teach and facilitate the practice of basic social skills in greetings, conversation, listening, and shifts in conversation to people with ASD. The results were positive, as users increased their knowledge and development of social skills. In addition, it has been indicated that this approach was well received by participants and caregivers. Ribeiro and Barbosa [35] developed a game called ComFiM, which aims to encourage communication between people with severe degrees of autism. The game was evaluated based on the perceptions of the interlocutors of each player and the communication intentions observed between the players to collaborate with each other and the results showed that the application positively influenced the communication intentions of the players.

The Emotions subcategory included studies that examined the development of skills such as the identification of facial emotions. Some studies from this subcategory are [36,37]. Romero [36] carried out a computer-based intervention to teach the recognition of emotions to students with communication and social skill deficits. All participants showed improvements when assessing and recognizing emotions on faces, but it was suggested that the effectiveness of the intervention should be tested in a larger population. Christinaki et al. [37] presented a serious game with a natural user interface (NUI) interaction that aims to teach young children with ASD to recognize and understand different facial emotions. The authors concluded that technological interventions with NUI improve the learning process and indicated that the emotional state of the players is directly related to their learning skills.

Additionally, the studies in the Interpersonal relationships subcategory emphasized individuals’ development of relationships. Some of the studies that were assigned to this subcategory are [38,39]. Boyd et al. [38] described how collaborative assistance technologies, such as the Zody collaborative game, can be used to facilitate social relationships in children with ASD. They discussed how design can foster three levels of social relationship, i.e., membership, partnership, and friendship, even without the help of adults. The results indicate that collaborative technologies provide support for the development of social skills at different levels of intimacy between players without a mediator during the intervention. Hourcade et al. [39] conducted an intervention with multitouch tablets with children with ASD to promote their social skills and help them develop their creativity, alter their interests, and be able to understand emotions. The result of the intervention was that it increased pro-social behaviors, such as collaboration, coordination, and interest in social activities, in children with ASD.

### 4.4. General Skills

Finally, the General Skills category included 29.79% of the studies. As this category referred to a range of topics, we defined only one subcategory, the General subcategory; some example studies are [40,41]. Backman et al. [40] investigated a method of evaluating children on the autism spectrum through computer games, which provide an objective, motivating, and safe evaluation of the participants. Although more research was recommended, the results showed that computer games have great potential in special education as an evaluation tool to clarify the difficulties associated with ASD. Hulusic and Pistoljevic [41] presented the initial development process of the LeFCA framework, which was used to teach children with ASD basic skills and concepts. LeFCA consists of four games that focus on developing basic skills (such as labeling, pointing and pairing in reference to visual and auditory stimuli) necessary for learning. Each of the participants was constantly motivated to play, and the skills learned could be extrapolated to new media or environments without the need for any training.

After reviewing all the studies and classifying them based on their learning topics, as shown in Table 5, we can see that there are a few studies that used modern and/or complex technologies, such as virtual reality or sensors. These technological approaches are interesting examples of how this area is developing in innovative ways.

Notably, most of the studies focused on teaching Social Skills, such as Emotions (12.77%), Communication (9.57%), and Language (14.89%), which are the most important areas that people with ASD have difficulties with.

**RQ2.**  **Which user experience and accessibility elements/methods are considered when analyzing the impact of technology on people with autism spectrum disorder?**

Although many of the studies suggested that accessibility and user experience are fundamental concepts for interventions with people who have ASD, these aspects were not treated with the importance that they should be.

Several of the studies that were reviewed from the pool of articles reported having used and/or considered user experience and/or accessibility, but most of these studies did not provide enough detail about the use of these concepts. Table A2 (available in Appendix A) shows a total of 23 studies that in some way used and/or provided "detail" on the use of these concepts in their research. We can see that the most recurrent terms used in the studies were user experience, usability, and accessibility.

For instance, many of the studies claimed to have focused on accessibility when developing touchscreen applications, such as [23,24,39,42,43]. However, the authors’ affirmations were not supported by empirical evidence or other details.

On the other hand, other studies such as [27,33] proposed the evaluation of the usability and/or user experience of the systems in future works. De Los Rios [33] suggested evaluating the usability of the application based on eye tracking. Schmidt and Beck [27] proposed the use of eye-tracking, electroencephalogram (EEG) scanning, and focus group interviews to evaluate the usability of the system.

Studies such as [28,40,42,44,45] aimed to evaluate usability and user experience based on post-intervention questionnaires with users, as well as with the people around them (such as their teachers or parents). These studies worked with control and test groups of children with and without ASD. Few studies indicated the number of subjects involved in the experiments: 14 in [42], 11 in [28], and 30 in [40]. Forty teachers were also involved in the experiment described in [42]. In addition to the questionnaires, Santarosa and Conforto [45] and Backman et al. [40] carried out methods such as focus groups in their interventions to be able to evaluate the usability and user experience.

Additionally, in studies such as those by Khowaja and Salim [46] and Naziatul et al. [47], the proposed systems were evaluated based on heuristic evaluations. In these studies, the authors adapted the heuristics proposed by Nielsen [48] to the contexts of their interventions. In both cases, three experienced evaluators assessed the system usability.

In addition, in the study by Vallefuoco et al. [49], a usability user test was carried out with 10 children aged between 5 and 12 under the methodology proposed by Moreno Ger [50] to evaluate the system, its usability, and the effectiveness of the customized elements developed to fulfill the objective of the study.

Finally, Caria et al. [22] worked with children with ASD between 16 and 22 years old, and Almeida et al. [51] worked with 40 children between 3 and 13 using the “System Usability Scale” (SUS) to evaluate the usability of their applications.

As we can see, few studies provided details about how they used concepts such as usability, user experience and accessibility, how these concepts were evaluated, and what kind of users were involved in their experiments. We think that it is important to consider all these concepts when developing new solutions.

**RQ3.**  **Which game elements are considered when using gamification or serious games in the education of people with autism spectrum disorder?**

Several of the identified studies described the use of game-based learning (mostly serious games), but they did not specify and/or provide details about the elements of the games that were used. However, a significant number of studies explicitly presented some game elements that allow these systems to be more attractive and engaging for users. In Table A3 (available in Appendix A), we can see the game elements used in the studies, where the most frequent elements were points, levels, and rewards. Brief definitions of the game elements, as presented by Werbach [10], are presented in Section 2.5.

For example, Vallefuoco et al. [49] analyzed a serious game that focused on improving math skills in children with ASD and for which one of the main elements was feedback. Likewise, Sorce et al. [18] used avatars in an application with Kinect to foster the interest of participants with ASD in digital representations of works of art, paintings, and sculptures. In addition, Romero [36] carried out a computer-based intervention with intrinsic rewards and points to teach the recognition of emotions. Similarly, Chen et al. [52] designed and developed a computer game with points and rewards to develop and evaluate emotional skills and conceptual comprehension skills (such as recognizing fruits) in children with autism spectrum disorder. Additionally, Harrold et al. [53] added to the concepts described above through the use of levels in CopyMe, a serious game for iPad, which provides children with ASD with a means to learn emotions through observation and mimics. In the same way, Sturm et al. [42] used stories in addition to rewards, points, and levels in a game with Kinect technology that aims to promote the recognition of emotions and encourage collaboration between people with ASD and their peers. Finally, Boyd et al. [38] described the use of Zody, as a collaborative assistance application, to teach social relations to children with ASD through the use of collaboration, points, levels, and rewards.

Most of the studies considered in this review did not explicitly identify which game elements they used in the development of their solutions. Even when they did, they did not give enough details on the effectiveness of the specific game elements. Although some authors claimed that their users were more engaged with the solutions they proposed, they did not provide empirical evidence to support such claims.

## 5. Conclusions and Future Work

Our systematic literature review focuses on analyzing the impact of technology on people with autism spectrum disorder based on research published during the last 10 years and available on the relevant scientific databases. The analysis shows an increase in the papers published on this topic over the years, which indicates an increasing research interest in the area. Interestingly, the highest percentage of the papers presented are case studies (74%). The studies were categorized into four categories: Conceptual Skills, Practical Skills, Social Skills, and General Skills. Studies that focus on Social Skills are predominant (36.17%).

Regarding RQ1, we observe that new research has focused on supporting children with ASD by using technologies such as virtual reality, augmented reality, virtual agents, sensors, and geolocation through educational games. These studies emphasize teaching different skills to people with ASD in educational contexts, with a higher percentage of studies focusing on Social Skills (36.17%) than on Conceptual (25.53%) or Practical Skills (8.51%), which shows a need for more research and development of new solutions for teaching such important topics. Exploring these alternatives and expanding the technological solutions to teach skills to people with ASD seem to be promising research topics.

The results related to RQ2 show that several studies mention that aspects such as user experience, usability, and accessibility are crucial when working with people with ASD. However, these aspects are usually not considered or validated in detail. Although the use of new technologies, such as EEG scanning and eye tracking in [27], to evaluate the usability of their systems is indeed interesting, studies have shown that brain activity may be negatively correlated with the Asperger questionnaire [54] and may be weaker for individuals with ASD when observing other people’s actions [55]. Future studies should be careful with the use of such technological approaches, as brain activity may be misleading when working with people with ASD, especially in tasks that require recognizing emotions from facial expressions or movements. We believe that user experience is important and that future studies should consider accessibility and usability tests to ensure positive experiences and comfort with the use of their solutions, as there is a lack of research that applies these concepts correctly and that provides details about the user groups that participate in interventions.

Regarding RQ3, we have observed in the literature that game elements are a good way to engage users with learning and enhance the effectiveness of teaching approaches for people with ASD, but our findings show that there is a lack of evidence about the effect of the use of game elements in gamification, e-learning, and serious game solutions. We believe that future studies should consider and validate the use of game elements. Werbach [10] highlighted that game elements are effective, have a positive relation with users’ engagement, and have been widely used with promising results.

We think that the use of technologies in conjunction with suitable game elements and user experience and accessibility design and evaluation are promising research topics related to teaching people with ASD.

## Figures and Tables

**Table 1:** Research questions for the systematic literature review.

ID	Research Question (RQ)
RQ1	In what way does the use of technology contribute to the education of people with autism spectrum disorder?
RQ2	Which user experience and accessibility elements/methods are considered when analyzing the impact of technology on people with autism spectrum disorder?
RQ3	Which game elements are considered when using gamification or serious games in the education of people with autism spectrum disorder?

**Table 2:** Search strings.

ID	Search Strings
SS1	(“Autism spectrum disorders” OR ASD OR Autism) AND (Accessibility OR “User experience”) AND (“game elements” OR gamification OR “Serious game” OR “game-based learning”)
SS2	(“Autism spectrum disorders” OR ASD OR Autism) AND (Accessibility OR “User experience”)
SS3	(“Autism spectrum disorders” OR ASD OR Autism) AND (“game elements” OR gamification OR “Serious game” OR “game-based learning”)

**Table 3:** Inclusion criteria.

ID	Inclusion Criteria
IN1	Studies published over the last 10 years, between January 2009 and June 2019.
IN2	Journal articles and conference papers.
IN3	Studies with a focus on autism spectrum disorder.
IN4	Studies related to the usage of technology.
IN5	Studies performed in an educative context or focused on teaching.

**Table 4:** Exclusion criteria.

ID	Exclusion Criteria
EX1	Studies with an exclusive medical focus or a focus on the diagnosis of autism spectrum disorder.
EX2	Studies that do not directly aim to help people with autism spectrum disorder but rather the people who work with them.
EX3	Studies that consider user experience and accessibility in contexts that do not involve the use of technology.

**Table 5:** Learning Topic.

Topic	Subtopic	Percentage by Subtopic	Percentage by Topic
Conceptual Skills	Language	14.89%	25.53%
	Money	1.06%	
	Colors	1.06%	
	Math	5.32%	
	Programming	2.13%	
	Science	1.06%	
Practical Skills	Healthcare	2.13%	8.51%
	Daily Living	3.19%	
	Transportation	3.19%	
Social Skills	Communication	9.57%	36.17%
	Emotions	12.77%	
	Interpersonal Relationships	13.83%	
General Skills	General	29.79%	29.79%

## Appendix A

**Table A1:** Identified Learning Topics.

Topic	Subtopic	Authors
Conceptual Skills	Language	Arciuli. J, Bailey. B. (2019) [13]
		Lin. C, Chang. S, Liou. W, Tsai. Y. (2013) [14]
		Wojciechowski. A., Al-Musawi. R. (2017) [15]
		Magaton. H, Bim. Silvia. (2017) [56]
		Mendonça. V, Coheur. L, Sardinha. A. (2015) [44]
		Wilson. C, Brereton. M, Ploderer. B, Sitbon. L. (2018) [57]
		Alvarado. C, Muñoz. R, Villarroel. R, et al. (2017) [58]
		Rasche. N, Pourcho. J, Wei. S, Qian. C.Z., Chen. V.Y. (2013) [59]
		Cunha. R.M., Barbosa. S.D.J. (2012) [60]
		Gomez. J, Jaccheri. L, Torrado. J.C, Montoro. G. (2018) [61]
		Khowaja. K, Salim. S.S, Al-Thani. D. (2018) [62]
		Khowaja. K, Salim. SS. (2019) [46]
		Khowaja. K, Al-Thani. D, Salim. SS. (2018) [21]
		Frutos. M, Bustos. I, Zapirain. B.G, Zorrilla. A.M. (2011) [63]
	Money	Caria. S, Paternò. F, Santoro. C, Semucci. V. (2018) [22]
	Colors	Tuğbagül Altan. N, Göktürk. M. (2018) [23]
	Math	Aziz. N.S.A, Ahmad. W.F.W, Hashim. A.S. (2016) [64]
		Naziatul. A.A, Wan. W.A, Ahmad. H. (2016) [47]
		Tashnim. A, Nowshin. S, Akter. F, Das. A.K. (2018) [24]
		Muñoz-Soto. R, Becerra. C, Noël. R., et al. (2016) [25]
		Aziz. N.S.A, Ahmad. W.F.W, Zulkifli. N.J.B. (2015) [65]
	Programming	Eiselt. K, Carter. P. (2019) [26]
		Schmidt. M, Beck. D. (2016) [27]
	Science	Eder. MS, Díaz. JML, Madela. JRS, Mag-usara. MU, Sabellano. DDM. (2016) [28]
Practical Skills	Healthcare	De Urturi. ZS, Méndez. A, García. B. (2011) [29]
		De Urturi. ZS, Méndez. A, García. B. (2012) [66]
	Daily Living	Pérez-Fuster. P, Sevilla. J, Herrera. G. (2019) [30]
		Santarosa. L.M.C, Conforto. D. (2016) [31]
		Lee. D, Frey. G, Cheng. A, Shih. PC. (2018) [67]
	Transportation	McKissick. B, Spooner. F, Wood. C.L, Diegelmann. K.M. (2013) [32]
		De Los Rios. P. C. (2018) [33]
		Rector. K. (2018) [68]
Social Skills	Communication	Milne. M, Raghavendra. P, Leibbrandt. R, Powers. D.M.W. (2018) [34]
		Bringas. J.A.S, León. M.A.C, Cota. I.E, Carrillo. A.L. (2016) [69]
		Hussain. A, Abdullah. A, Husni. H, Mkpojiogu. E.O.C. (2016) [70]
		Bernardini. S, Porayska-Pomsta. K, Smith. T.J. (2014) [17]
		Tang H.H, Jheng. C.M, Chien. M.E, Lin. N.M, Chen. M.Y. (2013) [71]
		El-Seoud. M.S.A, Karkar. A.G, Al Ja’am. J.M, Karam. O.H. (2015) [72]
		Baldassarri. S, Passerino. L, Ramis. S, Riquelme. I, Perales. FJ. (2018) [73]
		Cabielles-Hernández. D, Pérez-Pérez. J.-R, Paule-Ruiz. M, Fernández-Fernández. S. (2017) [74]
		Ribeiro. PC, Fox. AB. (2014) [35]
	Emotions	Romero. N.I. (2017) [36]
		Sturm. D, Kholodovsky. M, Arab. R, et al. (2019) [42]
		Papoutsi. C, Drigas. A, Skianis. C. (2018) [75]
		Lorenzo. G, Lledó. A, Pomares. J, Roig. R. (2016) [16]
		Leijdekkers. P, Gay. V, Wong. F. (2013) [76]
		Tinnunnem. S.G, Shah. G, Lahiri. U. (2017) [77]
		Harrold. N, Tan. C.T, Rosser. D, Leong. T.W. (2014) [53]
		Hyun. P.J, Abirached. B, Zhang. Y. (2012) [78]
		Castillo, T.A, Pérez de Celis. C, et al. (2016) [79]
		Almeida. LM, Silva. DPD, Theodório. DP, et al. (2019) [51]
		Cisnero. A.Q, Juárez-Ramírez. R., Figueroa. A.M. (2016) [80]
		Christinaki. E, Vidakis. N, Triantafyllidis. G. (2014) [37]
	Interpersonal Relationships	DiGennaro. F.D, Hyman. S.R, Hirst. J.M. (2011) [81]
		Boyd. L.E, Ringland. K.E, Haimson. O.L, Fernandez. H, Bistarkey. M, Hayes. G.R. (2015) [38]
		Muñoz. R, Morales. C, Villarroel. R, Quezada. A, De Albuquerque. V.H.C. (2019) [82]
		Rapp. A, Cena. F, Castald., R, Keller. R, Tirassa. M. (2018) [83]
		Sturm. D, Gillespie-Lynch. K, Kholodovsky. M. (2017) [84]
		Escobedo. L, Nguyen. D.H, Boyd. L.A, et al. (2012) [19]
		Hourcade. J.P, Bullock-Rest. N.E, Hansen. T.E. (2012) [39]
		Grossard. C, Grynspan. O, Serret. S, Jouen. A.L, Cohen. D. (2017) [85]
		Hughes. D.E, Vasquez E, Nicsinger. E. (2016) [86]
		Hani. H, Abu-Wandi. R. (2015) [87]
		Dehkordi. SR, Rias. RM. (2015) [88]
		Aziz. MZA, Abdullah. SAC, Adnan. SFS, Mazalan. L. (2014) [89]
		Jeekratok. K, Chanchalor. S, Murphy. E. (2014) [90]
General Skills	General	Backman. A, Mellblom. A, Norman-Claesson. E, Keith-Bodros. G, Frostvittra. M, Bölte. S, Hirvikoski. T. (2018) [40]
		Hong. E.R, Gong L.Y, Ninci. J, Morin. K, L.Davis. J, Kawaminami. S, Shi Y.G, Noro. F. (2017) [91]
		Still. K, May. R.J, Rehfeldt. R.A, Whelan R, Dymond. S. (2015) [92]
		Smith. K, Abrams. SS. (2019) [93]
		Cinquin. P.-A, Guitton. P, Sauzéon. H. (2019) [94]
		Jingga. F, Meyliana. Hidayanto. AN, Prabowo. H. (2019) [95]
		Constain. M. GE, Collazos O.C, Moreira. F. (2019) [96]
		Chen. J, Wang. G, Zhang. K, Wang. G, Liu. L. (2019) [52]
		Aziz. NSA, Ahmad. WFW, Hashim. AS. (2019) [97]
		Tsikinas. S, Xinogalos. S, Satratzemi. M, Kartasidou. L. (2018) [98]
		Çorlu. D, Taşel. Ş, Turan. S.G, Gatos. A, Yantaç. A.E. (2017) [99]
		Vallefuoco. E, Bravaccio. C, Pepino. A. (2017) [49]
		Tsikinas. S, Xinogalos. S, Satratzemi. M. (2016) [100]
		Wolff. M, Gattegno. M.P, Adrien. J.-L, Gabeau. C, Isnard. P. (2014) [101]
		Marchetti. E, Valente. A. (2015) [102]
		Alarcon-Licona. S, Loke. L, Ahmadpour N. (2018) [103]
		Sorce. S, Gentile. V, Oliveto. D, Barraco. R, Malizia. A, Gentile. A. (2018) [18]
		Mazon. C, Fage. C, Sauzéon. H. (2019) [104]
		Goosen. L. (2019) [105]
		Santarosa. L.M.C, Conforto. D. (2016) [45]
		Rahman. M.R, Naha. S, Roy. P.C, et al. (2011) [106]
		Silva Sandez. G, Rodriguez Miranda. F.P. (2018) [107]
		Silva. S.D, Neto. F.M.M, De Lima. R.M, De Macedo. F.T, Santo. J.R.S, Silva. W.L.N. (2017) [20]
		Kamaruzaman. N.N, Jomhari. N. (2015) [43]
		Tsikinas. S, Xinogalos. S. (2019) [108]
		Helmi Adly. MN, Faaizah. S, Naim. CP. (2014) [109]
		Boucenna. S, Narzisi., A, Tilmont. E, et al. (2014) [110]
		Hulusic. V, Pistoljevic. N. (2012) [41]

**Table A2:** Identified User Experience Concepts.

Authors	Identified Concepts
Backman. A, Mellblom. A, Norman-Claesson. E, Keith-Bodros. G, Frostvittra. M, Bölte. S, Hirvikoski. T. (2018) [40]	Usability
Romero. N.I. (2017) [36]	Accessibility
Sturm. D, Kholodovsky. M, Arab. R, et al. (2019) [42]	Usability
Constain. M. GE, Collazos O.C, Moreira. F. (2019) [96]	Usability, accessibility
Muñoz. R, Morales. C, Villarroel. R, Quezada. A, De Albuquerque. V.H.C. (2019) [82]	Usability
Caria. S, Paternò. F, Santoro. C, Semucci. V. (2018) [22]	Usability, accessibility
Milne. M, Raghavendra. P, Leibbrandt. R, Powers. D.M.W. (2018) [34]	Usability
Wojciechowski. A., Al-Musawi. R. (2017) [15]	Usability
Vallefuoco. E, Bravaccio. C, Pepino. A. (2017) [49]	Usability
Naziatul. A.A, Wan. W.A, Ahmad. H. (2016) [47]	Usability
Sorce. S, Gentile. V, Oliveto. D, Barraco. R, Malizia. A, Gentile. A. (2018) [18]	Usability, accessibility
De Los Rios. P.C. (2018) [33]	Usability, accessibility
Mendonça. V, Coheur. L, Sardinha. A. (2015) [44]	User experience
Rector. K. (2018) [68]	Accessibility
Tang H.H, Jheng. C.M, Chien. M.E, Lin. N.M, Chen. M.Y. (2013) [71]	Usability, accessibility, user experience
Muñoz-Soto. R, Becerra. C, Noël. R., et al. (2016) [25]	Usability, accessibility, user experience
Santarosa. L.M.C, Conforto. D. (2016) [45]	Usability, accessibility
Khowaja. K, Salim. SS. (2019) [46]	Usability
Almeida. LM, Silva. DPD, Theodório. DP, et al. (2019) [51]	Usability, accessibility
Lee. D, Frey. G, Cheng. A, Shih. PC. (2018) [67]	Usability, accessibility, user centered
Cabielles-Hernández. D, Pérez-Pérez. J.-R, Paule-Ruiz. M, Fernández-Fernández. S. (2017) [74]	Accessibility
Schmidt. M, Beck. D. (2016) [27]	Usability
Eder. MS, Díaz. JML, Madela. JRS, Mag-usara. MU, Sabellano. DDM. (2016) [28]	Usability

**Table A3:** Identified Game Elements.

Authors	Game Elements Referenced
Romero. N.I. (2017) [36]	Points, intrinsic rewards
McKissick. B, Spooner. F, Wood. C.L, Diegelmann. K.M. (2013) [32]	Achievement, levels
Lin. C, Chang. S, Liou. W, Tsai. Y. (2013) [14]	Points
Bistarkey. M, Hayes. G.R. (2015) [38]	Points, levels, rewards, collaboration
Sturm. D, Kholodovsky. M, Arab. R, et al. (2019) [42]	Points, levels, rewards, narrative, collaboration
Muñoz. R, Morales. C, Villarroel. R, Quezada. A, De Albuquerque. V.H.C. (2019) [82]	Levels
Chen. J, Wang. G, Zhang. K, Wang. G, Liu. L. (2019) [52]	Points, levels, rewards
Aziz. NSA, Ahmad. WFW, Hashim. AS. (2019) [97]	Levels
Caria. S, Paternò. F, Santoro. C, Semucci. V. (2018) [22]	Levels
Milne. M, Raghavendra. P, Leibbrandt. R, Powers. D.M.W. (2018) [34]	Feedback, rewards
Sturm. D, Gillespie-Lynch. K, Kholodovsky. M. (2017) [84]	Levels, collaboration
Vallefuoco. E, Bravaccio. C, Pepino. A. (2017) [49]	Feedback
Bringas. J.A.S, León. M.A.C, Cota. I.E, Carrillo. A.L. (2016) [69]	Points, levels, feedback, rewards
Lorenzo. G, Lledó. A, Pomares. J, Roig. R. (2016) [16]	Avatars
Bernardini. S, Porayska-Pomsta. K, Smith. T.J. (2014) [17]	Avatars
Sorce. S, Gentile. V, Oliveto. D, Barraco. R, Malizia. A, Gentile. A. (2018) [18]	Avatars
Magaton. H, Bim. Silvia. (2017) [56]	Levels
Tashnim. A, Nowshin. S, Akter. F, Das. A.K. (2018) [24]	Rewards
Alvarado. C, Muñoz. R, Villarroel. R, et al. (2017) [19]	Points, levels
Muñoz-Soto. R, Becerra. C, Noël. R., et al. (2016) [25]	Points, levels
Escobedo. L, Nguyen. D.H, Boyd. L.A, et al. (2012) [19]	Points, levels, rewards
Harrold. N, Tan. C.T, Rosser. D, Leong. T.W. (2014) [53]	Points, levels, rewards
Khowaja. K, Salim. SS. (2019) [46]	Achievement, points, levels, rewards
Almeida. LM, Silva. DPD, Theodório. DP, et al. (2019) [51]	Points, levels, avatars, feedback
Lee. D, Frey. G, Cheng. A, Shih. PC. (2018) [67]	Rewards
Baldassarri. S, Passerino. L, Ramis. S, Riquelme. I, Perales. FJ. (2018) [73]	Points, levels
Hughes. D.E, Vasquez E, Nicsinger. E. (2016) [86]	Achievement, points, avatars
Schmidt. M, Beck. D. (2016) [27]	Team
Eder. MS, Díaz. JML, Madela. JRS, Mag-usara. MU, Sabellano. DDM. (2016) [28]	Levels
Dehkordi. SR, Rias. RM. (2015) [88]	Points, levels, rewards
Ribeiro. PC, Fox. AB. (2014) [35]	Levels, collaboration
De Urturi. ZS, Méndez. A, García. B. (2012) [66]	Levels, avatars
